# On the Operational Conditions’ Effect on the Performance of an Anion Exchange Membrane Water Electrolyzer: Electrochemical Impedance Spectroscopy Study

**DOI:** 10.3390/membranes13020192

**Published:** 2023-02-03

**Authors:** Irina V. Pushkareva, Maksim A. Solovyev, Sergey I. Butrim, Margarita V. Kozlova, Dmitri A. Simkin, Artem S. Pushkarev

**Affiliations:** 1 National Research Center “Kurchatov Institute”, 1 Kurchatov sq., Moscow 123182, Russia; 2 Department of Chemistry and Electrochemical Energy, National Research University “Moscow Power Engineering Institute”, 14 Krasnokazarmennaya str., Moscow 111250, Russia

**Keywords:** anion exchange membrane, water electrolysis, operation conditions, supporting electrolyte, electrochemical impedance spectroscopy, stray impedance

## Abstract

The performance of an anion exchange membrane water electrolyzer under various operational conditions (including voltage, KOH-supporting electrolyte concentration, and flow rate) is studied using conventional time-domain technics and electrochemical impedance spectroscopy (EIS). The water electrolyzer EIS footprint, depending on the variation in operational conditions, is studied and discussed, providing valuable data on the faradaic and non-faradaic processes in MEA, considering their contribution to the total polarization resistance. The distribution of the AEMWE cell voltage contributions is valuable to accessing the key directions in the system performance improvement.

## 1. Introduction

Recently, water electrolysis has gained much attention, since it allows the relatively quick and convenient production of “green” hydrogen from only water and electricity. In particular, membrane electrolysis offers a sustainable solution to produce hydrogen, which may be coupled with widespread intermittent renewable energy sources (e.g., wind and solar) as well as with nuclear power, allowing for effective grid balancing [1]. There are two main technologies according to which membrane electrolyzer could be designed: proton exchange membrane (PEM) technology [2] and anion exchange membrane (AEM) technology [3].

AEM water electrolyzers have shown significant technological development thus far [3,4,5]. They have all alkaline water electrolysis advantages: inexpensive, non-noble-metal-based catalysts and stack materials (in contrast to the acidic polymer electrolyte membrane electrolysis), easy handling due to the relatively low temperatures, and lower alkaline solution concentrations (which could be even replaced with pure water or a K_2_CO_3_ solution [6]). The high performance of proton exchange membrane (PEM)-based systems is ensured by the zero-gap cell approach, with minimal electrode distance and ohmic loss [7]. The use of the AEM allows for the application of a similar concept, preserving the advantages of the classic alkaline technology, valorizing the potential of the AEM water electrolysis technology.

It is desirable to operate the electrolyzer at low electrolyte concentrations or only with water [8] to suppress the degradation of the materials. Some AEMs are not stable enough in even 1 M of KOH, and catalysts (especially at the anode where the oxygen evolution occurs) are prone to dissolve [9]. However, the electrolytes that have been most commonly used in AEM electrolyzers are hydroxide solutions (KOH and NaOH) [4]. The hydroxide-based liquid electrolyte provides many benefits with respect to electrolyzer performance: (1) a lowered HFR; (2) significantly increased OER kinetics; (3) marginally increased HER kinetics and (4) a facilitated ion-transport in ionomer by an increased ionic conductivity [10].

The liquid electrolyte may be circulated through both the anode and cathode compartments [11,12], the anode compartment only [13,14], and even the cathode compartment only [15]. The circulation of the liquid electrolyte through both electrode compartments is beneficial from the performance point of view, though rather high performance could be achieved with only the anode being fed with the alkaline solution [4]. On the other hand, the KOH circulation through both compartments ensures the full AEM humidification and saturation with OH^−^ charges. According to Park et al. [16], feeding both compartments enhanced the ionic conductivity of AEM and directly supplied hydroxide ions at the anode, resulting in an enhanced performance. However, not many studies have been performed thus far on the hydroxide-based liquid electrolyte feeding. Even when symmetrical electrolyte feeding to both compartments is used, its flow rate needs to be optimized, and its effect on the kinetics and cell resistance needs to be revealed. Currently, “operando” characterization methods are urgently needed to probe the water electrolysis process during operation in real time [17]. EIS is among the promising approaches, as it allows for the non-invasive “in-situ” measurement of an electrochemical system’s impedance [18] and has been widely applied in the R&D of Li-ion batteries [19], fuel cells [20], and others [21,22].

The EIS approach allows certain processes occurring in an electrochemical system to be distinguished considering the differences in their relaxation time [18]. The EIS measurements of MEAs usually perform at different values of voltage/current density under different operational conditions (i.e., temperature, supporting electrolyte type, concentration, and feeding and flow rate), so there is no well-established approach to study and analyze the components observed in the Nyquist/Bode plots [23,24]. EIS spectra are commonly analyzed using equivalent circuits, using the prior experience to find a circuit comprising a finite set of elementary (resistors, capacitors, and inductors) and generalized electrical elements/circuits that can match the data [25]. This approach is rather attractive due to its simplicity instead of its drawbacks, such as the ambiguity and uncertainty in interpreting EIS data based on ECMs [26].

The water electrolyzer EIS footprint includes from one to three R/CPE components [23,27,28,29], though their relation to certain processes in the MEA is not clear yet. R/CPE components might be assigned to both faradaic (HER and/or OER) [30] and non-faradaic processes (such as electrode–electrolyte interface resistance [31] or gas/liquid transport [27,32]). Some physical models to describe both the faradaic and non-faradaic processes in water electrolyzer have been proposed so far [33,34].

In the present work, to better investigate the operational conditions affecting the AEM water electrolyzer’s performance, MEAs based on the widespread, commercially available Sustainion^®^ membrane were fabricated using the CCS approach and were studied by the EIS using the proposed equivalent circuit. The accurate evaluation of the MEA ohmic (so-called “high-frequency resistance”) and charge transfer resistance was ensured by the proposed stray impedance subcircuit and its evaluation. The effect of cell voltage, KOH concentration, and flow rate on the performance of an AEM electrolyzer and its EIS footprint were analyzed and discussed considering non-faradaic contributions to the MEA polarization resistance.

## 2. Experimental

The commercially available Sustainion^®^ X37-50 (Dioxide Materials, Boca Raton, FL, USA) [12] and Ni foam (porosity 90–95%, 110 PPI) (Ohmliberscience, St. Petersburg, Russia) were used as an AEM and in porous transport layers (PTL), respectively. MEAs were prepared according to the catalyst-coated substrate (CCS) approach: the membrane was sandwiched between catalyst-coated electrodes. To ensure the MEA’s mechanical stability and flatten its surface, the foam-based electrodes (2.7 × 2.7 cm) were pressurized so that their final thickness was ca. 0.3 mm. To increase the MEA integrity and develop the catalyst layer (CL)/AEM interface, the microporous sublayer [27], composed of Ni powder, was sprayed over the PTL surface (Ni powder with a particle size of ca. 3–20 µm and a loading of ca. 10 mg cm^−2^). Pt/C (PM40, PROMETHEUS R&D, LLC, Rostov-on-Don, Russia) [35] and NiO [36] were used as cathode and anode catalysts, respectively. The Pt loading was ca. 0.8 mg cm^−2^, and the NiO loading was ca. 3 mg cm^−2^. First, cathode and anode catalyst slurries (so-called “inks”) were obtained by mixing the powder, Nafion^®^ (Ion Power, New Castle, DE, USA) binder, and isopropanol. CLs were then obtained via air-spraying catalytic inks over the PTL surfaces [37,38]. The MEAs were formed by self-assembly [37,39] in the test cell: the PTLs and the membrane were fixed in between two stainless steel half-cells equipped with flow-fields (parallel channels). MEA preconditioning was performed by keeping the cell at a temperature of 60 °C for 2 h to ensure the tight binding of the MEA components, and different voltage steps were consequently applied until the reproducible polarization behavior of the MEA was obtained. The cell was operated at atmospheric pressure on both sides.

**Figure 1:**
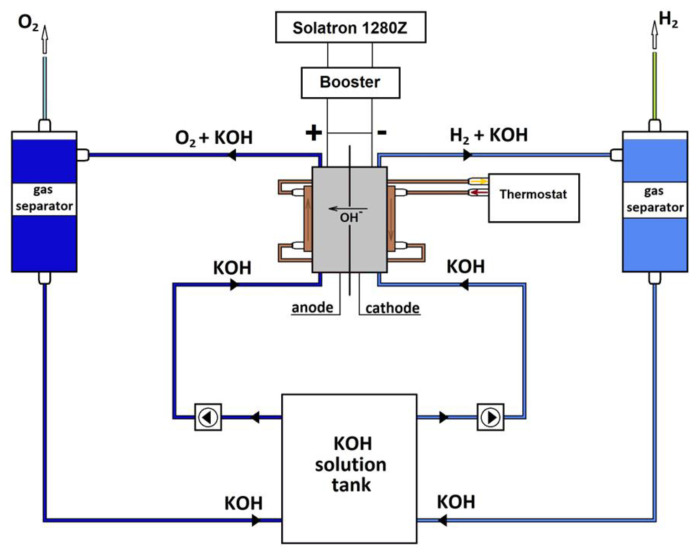
The test bench, which was used to evaluate MEA performance and EIS.

The Nafion binder was chosen for its good stability in alkaline solutions and its small effect on the MEA conductivity, as the hydroxide supply is established by the supporting electrolyte [10,40]. Recently, we reported an outstanding AEM water electrolyzer performance using different AEMs and Nafion-bonded electrodes based on non-noble catalysts [11]. The performance of AEMWE MEAs were evaluated using the SI 1280 potentiostat (AMETEK, Inc., Berwyn, PA, USA), which was equipped with a power booster 12 V/20 A (AMETEK, Inc., Berwyn, PA, USA) to capture the polarization curves and EIS (Figure 1). Polarization curves were measured in potentiodynamic mode (1.4–2.05 V) at a sweep rate of ca. 1 mV s^−1^. Such an approach provides only a quasi-steady-state conditions, but is a compromise considering the low time consumption of the measurements [11,41]. Both the anode and cathode compartments of the test cell were fed with a supporting electrolyte of 0.1–1.0 mol l^−1^ KOH solution with the same flow rate, which varied in the range of 0.1–10 mL min^−1^. The outlet-supporting electrolyte flows were mixed in the tank with magnetic stirring to ensure a steady KOH concentration in the system. The anode and cathode separators were necessary to avoid the loss of supporting electrolyte with gases released.

The EIS measurements were performed under potentiostatic control in a frequency range between 20 kHz and 100 mHz by frequency sweeping in the single-sine mode. It is important to note that points at 100 and 200 Hz systematically deviated from typical behavior. They are suggested as artefacts and should therefore be excluded during the analysis [42]. The cell equilibration period of at least 300 s was included before the EIS measurement. An AC signal amplitude of 10 mV was used in all measurements to keep the cell response stable and linear [18,42]. EIS data were mainly processed using the open-access software pyZwx 1.0.3 [43], and the Lin-KK [44] software was used to evaluate the quality of the data. The results of the Kramers–Kronig validity testing in the form of the residuals between the fitting and raw data are provided in Figure S1 in Supplementary Materials. These residuals were low enough (mainly less than 2%) to suggest the validity of the impedance spectra in compliance with the linearity and time invariance criteria [45]. It is important to note that a Lin–KK analysis was applied to raw spectrums, and the artefacts at 100 and 200 Hz as well as the stray impedance contributed considerably to the residual values. The high accuracy of the fitting is proved by the rather low values of the reduced chi square factor (provided by pyZwx after fitting): 5–50 × 10^−5^.

## 3. Results and Discussion

EIS provides a direct measurement of important parameters of water electrolysis MEA, such as the ohmic and polarization resistances. The so-called “high-frequency resistance” (HFR) and “low-frequency resistance” (LFR) [46] could be simply extracted from the EIS spectra without a complex, nonlinear least-square fitting. The HFR value is taken at high frequencies (1–25 kHz [7,47]) as the real impedance of the Nyquist plot interception with the axis, and is typically assigned to the MEA ohmic resistance. However, the high-frequency artefacts (or stray impedance [19,48,49]) also appear at the same frequencies, so the HFR value could be strongly affected and may not accurately represent the MEA ohmic resistance. Further, the LFR value could be evaluated as the difference between the HFR and the Nyquist plot interception with the Re axis at lower frequencies [46]. If there are no mass-transfer limitations (typically appearing at low frequencies [32]) or strong, pseudo-inductive behavior [50], both of which may considerably affect the system, the LFR could be considered to be MEA polarization resistance, R_ct_. Otherwise, the CNLS fitting with a pre-chosen model should be performed, and charge transfer resistances (“R_i_” from all charge transfer components, R/CPE) should be summarized.

In the present study, the MEA EIS footprint was strongly affected by the high-frequency stray impedance [51,52] originating from the equipment (booster) and/or the cell connection issues. It appears as a high frequency “hook” with a positive Im part. Details on the approach to considering this stray impedance and eliminating its effect on the MEA spectrum analysis are given in SI.

**Figure 2:**
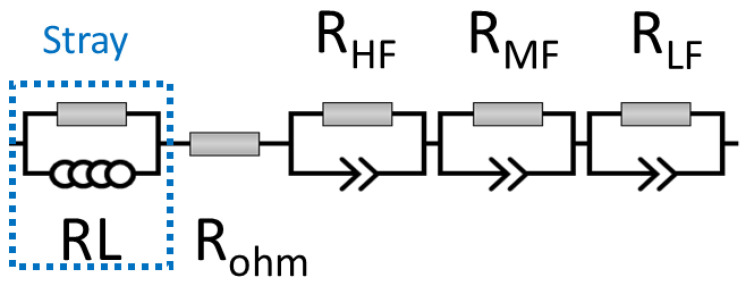
The equivalent circuit used to analyze the EIS data.

Recently, we proposed an equivalent circuit with stray inductivity, MEA ohmic resistance, and three R/CPE components (denoted as high-, medium-, and low-frequency components) [53]. Due to the use of non-PGM catalysts for both HER and OER and a limited voltage range, these R/CPE elements were assigned to non-faradaic, high-frequency features: HER and OER, respectively. The modified approach was used in the presented study, as different hardware and catalysts were used. Figure 2 shows the equivalent circuit applied in the presented study, considering the stray impedance subcircuit (Figure S2). Due to the wider voltage range under study, the low-frequency component, R_LF_, which was assigned to the mass-transfer losses, appeared at voltages ≥1.8 V [54]. Ohmic resistance, R_ohm_, comprises both the ionic and ohmic resistances of the MEA components (AEM, CLs, PTLs, etc.). The sum of R_HF_ and R_MF_ provides charge transfer resistance, R_ct_. The CPE element is related to the non-ideal capacitive behavior of the respective interfaces, as well as the non-uniform distribution of the catalyst’s active sites [55]. The applicability of the proposed equivalent circuit as well as the possible processes assigned to certain R/CPE components are discussed later.

The higher current density operation of electrolyzer is beneficial in terms of the capital cost of electrolysis (higher hydrogen production rate) [56]. On the other hand, the operation of electrolysis at higher current reduces the voltage efficiency and increases component degradation [57] leading to an increase in the generated hydrogen cost. Thus, in the present study, the effect of current density on the MEA resistance components was studied in a wide range of current densities, up to 2.5 A cm^−2^. It is important to note that the EIS measurements were performed in potentiostatic mode at certain values of the cell voltage: 1.4–2.0 V, stabilized for at least 300 s. The polarization curves correlate very well with the currents obtained during the stabilization step before EIS.

**Figure 3:**
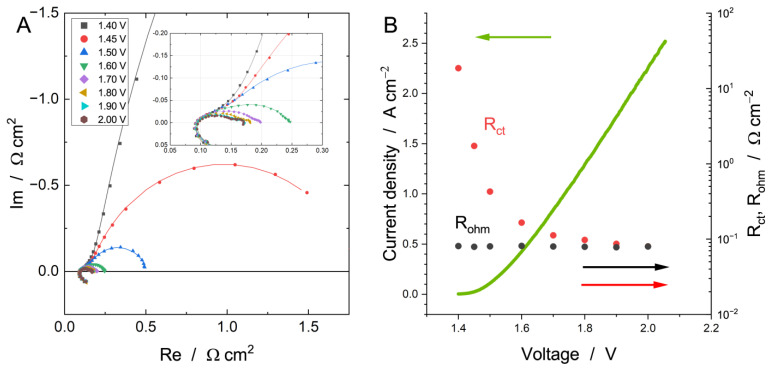
Nyquist plots (**A**) of AEM water electrolyzer measured at different cell voltage (60 °C, 1.0 M KOH, flow rate of 3 mL min^−1^). The fitting was performed according to equivalent circuit given in Figure 2. The dependencies of R_ohm_ and R_ct_ on the cell voltage are given in section (**B**) (the right *Y*-axis for R_ohm_ and R_ct_ is scaled logarithmically).

Typical Nyquist plots of water electrolysis are provided in Figure 3A and consist of the stray impedance (the low-frequency “tail” with positive imaginary impedance) and few arcs located above the real axis. These arcs are large at lower voltages. They decrease with an increase in the current density (voltage) and are related to the cell polarization. According to the typical water electrolyzer polarization curve (provided in Figure 3B) [58], the increase in current density leads to the increase in cell voltage (due to the HER and OER overvoltage increasing), suggesting a decrease in charge transfer resistance. At a voltage ≥1.8 V, the third arc appears at low frequencies, suggesting some mass-transfer limitations.

The polarization curve of the MEA operated at 60 °C using 1 M KOH is provided in Figure 3B with the values of R_ohm_ and R_ct_. Typically, a polarization curve (or “V-I curve”) consists of three main regions: a low current density region with kinetic control (usually up to 100 mA cm^−2^, Tafel region [37,59]), a medium current density region with ohmic resistance control (0.1–2… A cm^−2^, depending on the hardware, PTLs, catalysts, and others), and a high current density region with mass-transfer losses appearing (for instance, due to limited gas removal [60]). The latter is absent in our case, which is likely due to the very high porosity of the PTLs, which allow gases to leave compartments easily [37]. The MEA ohmic resistance, R_ohm_, falls within a narrow range of ca. 78–82 mΩ cm^−2^ and is rather independent of the cell voltage. It is mainly described by the usage of 1 M KOH of supporting electrolyte with a constant flow rate, which provides free charges to ensure a high ion conductivity of the membrane and the CLs [37]. The obtained AEM water electrolyzer performance was rather high in comparison with the literature (Table S1), considering that a non-noble OER catalyst (NiO) was used. In contrast, the charge transfer resistance, R_ct_, drops logarithmically with the increasing voltage (current density), indicating their relation to the HER and OER.

**Figure 4:**
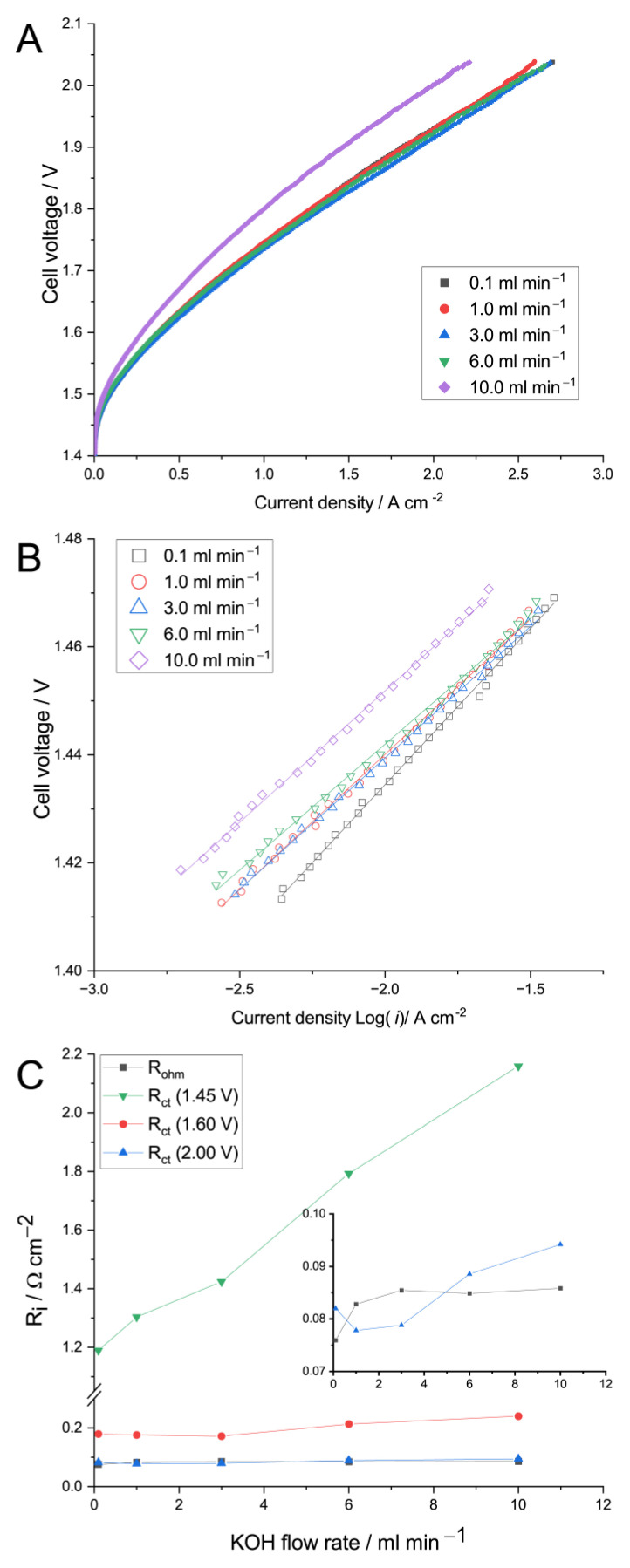
Polarization (**A**) and Tafel (**B**) curves of the MEA studied at different 1 M KOH-supporting-solution flow rates through both anode and cathode compartments at 60 °C. The effect of KOH flow rate on the R_i_ values (**C**) obtained at 1.45; 1.6, and 2 V are also provided.

We also evaluated the effect of the supporting electrolyte flow rates (0.1–10 mL min^−1^ or 0.014–1.4 mL min^−1^ cm^−2^) to determine the optimal 1 M KOH flow rate. It is important to note that in most of the studies that employed the Sustainion^®^ membrane, the two-compartment-feeding approach was used [11,12,40,61,62]. Therefore, high membrane conductivity can be ensured only when the membrane is sufficiently humidified (on both sides) and saturated with OH^−^. Thus, a simultaneous KOH-pumping scheme with the same flow rate on the anode and cathode was chosen. Figure 4 shows the polarization (A) and Tafel (B) curves of the MEA studied at different 1 M KOH-supporting-solution flow rates. Typically, the Tafel slope can be obtained from the Tafel plot (U–iR~log(*i*)), and the lower current density region (1–100 mA cm^−2^) is taken [63] as the transport overpotential is suggested to be negligible. The Tafel plots are linear (due to the non-linear behavior of the polarization curve at lower current density) until the current density reaches a value of ca. 0.1 A cm^−2^, suggesting a water electrolysis process governed by the hydrogen evolution (HER) and oxygen evolution (OER) reaction kinetics. It is important to note that the Tafel slope calculated using the MEA polarization curve consists of contributions of both the cathode and the anode [37,64].

**Figure 5:**
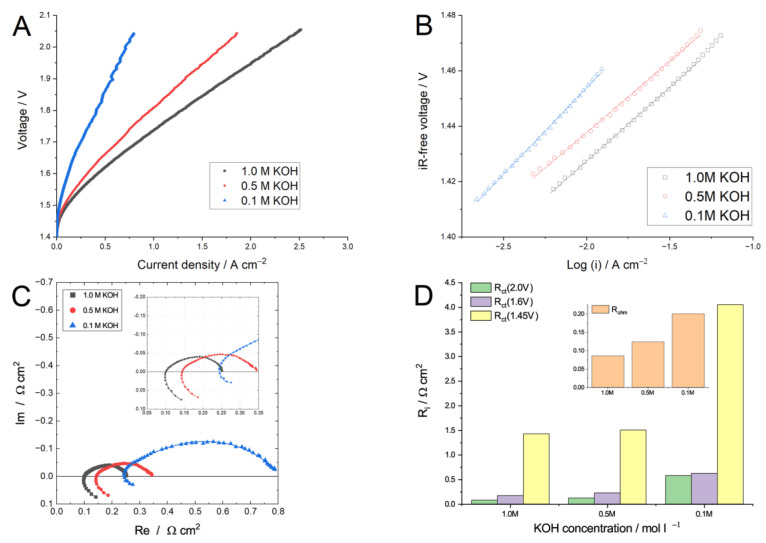
Polarization (**A**) and Tafel (**B**) curves of the MEA studied with different KOH concentration solutions pumped through both anode and cathode compartments at 3 mL min^−1^ at 60 °C. The effect of KOH molarity on the Nyquist plots (**C**), measured at 1.6 V, and the R_i_ values (**D**), obtained after EIS data fitting at 1.45; 1.6, and 2 V, are provided.

The polarization curves of the MEA suggest a rather weak effect of the KOH flow rate up to 6 mL min^−1^: the current density at 2.0 V is ca. 2.41, 2.42, 2.47, 2.45, and only 2.0 A cm^−2^ for flow rates of 0.1, 1.0, 3.0, 6.0, and 10.0 mL min^−1^, respectively. Obviously, at flow rates >6 mL min^−1^, the MEA performance considerably deteriorated. According to Figure 4B, the Tafel slope takes a value of 57.8, 50.0, 48.6, 46.6, and 48.7 mV dec^−1^ for flow rates of 0.1, 1.0, 3.0, 6.0, and 10.0 mL min^−1^, respectively. Its increase at the lowest flow rate could be ascribed to the limited local accessibility of OH^−^ ions in the AEM/CL interface. This conclusion is in a good agreement with the Tafel slope for the MEA operated with a lowered KOH concentration (see below). It is important to note that the kinetic current of the MEA is rather similar for low flow rates, in the range of 0.1–6.0 mL min^−1^, but increases with a further increase in the flow rate. Figure 4C shows the R_ohm_ and R_ct_ values obtained at 1.45, 1.6, and 2 V for different 1 M KOH flow rates. Due to the practical independence of R_ohm_ on the cell voltage, the R_ohm_ values in Figure 4 and Figure 5 were taken as a mean value among the voltage range under study. The R_ohm_ was also found to be independent of the KOH flow rate at 1.0–10 mL min^−1^ (0.14–1.4 mL min^−1^ cm^−2^). The nature of rather significant R_ohm_ drop switching from 1 to 0.1 mL min^−1^ (∆R_ohm_ = 8%) is unclear and could be related to any unsteady MEA behavior at such a low flow rate.

The charge transfer resistance values evaluated at different voltages are provided in Figure 4C to unravel the effect of the flow rate on the electrode’s performance. The R_ct_ (1.45) increased with the increase in the KOH flow rate, suggesting a slight deterioration of the HER and/or OER kinetics, which is in a good agreement with Tafel curves (Figure 4B).

The R_ct_ (1.6) and R_ct_ (2.0), measured at practical voltages for hydrogen production, pass through their minimum at 1–3 mL min^−1^, suggesting the optimal conditions between the OH^−^ availability at the AEM/CL interface and the produced gas removal, which should be enhanced due to the increased flow rate. Finally, the flow rate of 3 mL min^−1^ was chosen as an optimal value due to the maximum current density (Figure 4A), minimal R_ct_ (1.6), and a trade-off between other parameters, i.e., R_ohm_, R_ct_ (1.45), and R_ct_ (2.0).

Park et al. [16] showed that at flow rate of 2.5 mL min^−1^ (0.5 mL min^−1^ cm^−2^), sufficient OH^−^ reactant was supplied to the anode, saturating the cell performance. The performance was not enhanced further with an increased flow rate of up to 10 mL min^−1^ (2.0 mL min^−1^ cm^−2^). Therefore, 2.5 mL min^−1^ is considered the optimal reactant flow rate. Rather high flow rates were applied in Ref. [23] for a PBI-based AEMWE, with an anode-only 1 M KOH feeding approach: 0–110 mL min^−1^ (0–22 mL min^−1^ cm^−2^). Authors reported a significant improvement in the AEMWE performance when the flow rate increased up to 8 mL min^−1^ cm^−2^, which was provided by the decrease in ohmic and charge transfer resistances. It is important to note that a further increase in flow rate leads to an increase in the OER charge transfer resistance and ohmic resistance. Authors suggested that a higher flow rate rapidly removes the OH^−^ ions, thereby reducing the available reaction time for OER and subsequently leading to a lower availability of OH^−^ ions for the catalyst. These suggestions are in good agreement with our data.

Another important operating condition is the electrolyte solution concentration, as the water electrolyzer cell performance is highly dependent on pH [6,64]. In pure water, without the additional transport pathways enabled by the liquid electrolyte, the ECSA at the catalyst/liquid-electrolyte interface may be not effectively utilized [10]. The usage of 1 M of KOH solution is suggested as a compromise between superior AEM water electrolyzer performance and AEM stability, as it provides a rather high membrane and catalytic layer conductivity [9], and the contribution of the binder (basically anion exchange polymers [65], as PTFE [66] and Nafion [11] were suggested) is not too significant. However, a decrease in KOH concentration down to 0.1 M leads to the significant deterioration of the AEM water electrolyzer performance (Figure 5). Figure 5A shows polarization curves obtained at 60 °C with the 3 mL min^−1^ supporting electrolyte flow rate. A decrease in KOH molarity from 1.0 to 0.1 M leads to a more than 3-fold decrease in the current density at 2.0 V, dropping from 2.25 to 0.71 A cm^−2^. Considering the CCS concept of the MEA in the study, an increase in the supporting electrolyte pH improves the catalyst utilization due to the expanded catalyst–electrolyte interfacial area [9] and the improved intrinsic kinetics of the catalysts [13,67]. In Figure 5C, the Tafel plots for the variation of KOH concentrations are provided. One can see that the kinetic current decreases with a decrease in the KOH molarity (the plot goes upward), which is in line with recent reports on the activity of HER and OER catalysts [67]. Though the Tafel slope change is moderate, it fell in a tiny range of 52–62 mV dec^−1^, suggesting the same HER/OER mechanism in the whole KOH concentration range. According to Figure 5C, the decrease in KOH molarity deteriorates the MEA ohmic resistance (Nyquist plot shifts to the right) and leads to a significant increase in charge transfer resistance: the arc diameter (LFR) increases, suggesting a slower charge transfer. Figure 5D shows the comparison of the R_ohm_ and R_ct_ values measured at different voltages. Obviously, the switch to 0.1 M of KOH leads to a more than 2-fold increase in the MEA ohmic resistance and to a more than 3-fold increase in the MEA charge transfer resistance (from 0.17 to 0.62 Ω cm^−2^, measured at 1.6 V). This indicates that the pH affects the AEM electrolyzer performance not only through iR effects, but also through HER and OER catalysis. It is important to note that the switch to a 0.5 M KOH solution concentration leads to a moderate decrease in the cell performance: the current density decreases ca. 24%, so the 0.5 M KOH supporting electrolyte should at least be used to achieve high performance. Vincent et al. reported [23] a more than 3-fold increase in the PBI-based MEA resistance from 0.09 to 0.334 Ω, suggesting a decrease in the ion concentration within the membrane and a possible contribution of Osmotic deswelling effects.

The method of EIS could be used to unravel the particular yield of each process occurring in MEA to an overall polarization resistance, depending on operational conditions. Due to the different relaxation times of different process (e.g., ohmic resistance, electrode/electrolyte charge transfer, HER, OER, and mass transport) it is possible to separate them in the frequency domain in contrast to the time domain technique (e.g., the polarization curves given in Figure 3A). However, distinguishing the cathode and anode is not straightforward. For instance, a circuit with only two R/CPE elements displaying the cathode and the anode could be used [23,68], though such an approach could be effective if you do not have significant non-faradaic contributions at high and medium frequencies, and the rate difference between the HER and OER are large enough to avoid their significant overlap. For PEM water electrolysis, there is a significant difference between the HER and OER rates, so the contribution of HER could be suggested to be negligibly small [69]. In the present study, a Pt/C based cathode catalyst with relatively high Pt loading was used, suggesting a negligibly small contribution from the HER to the charge transfer resistance. In contrast, non-noble catalysts are used in our recent work [53], and the contribution of the NiFe_2_O_4_-based anode and the NiFeCo-based cathode were suggested to be comparable. Two separate R/CPE elements were assigned to their charge transfer resistance contribution. The third R/CPE element was proposed for high-frequency contributions from a non-faradaic process, the origin of which is still unclear and is discussed in ref. [53]. It is important to note that this high-frequency contribution is independent of the cell voltage.

In the present study, we proposed an equivalent circuit (Figure 2) consisting of three R/CPE elements assigned as follows: high-frequency non-faradaic charge transfer, OER charge transfer (HER is too fast, so its contribution is suggested to be negligible), and mass-transfer (appearing at voltage ≥1.8 V).

**Figure 6:**
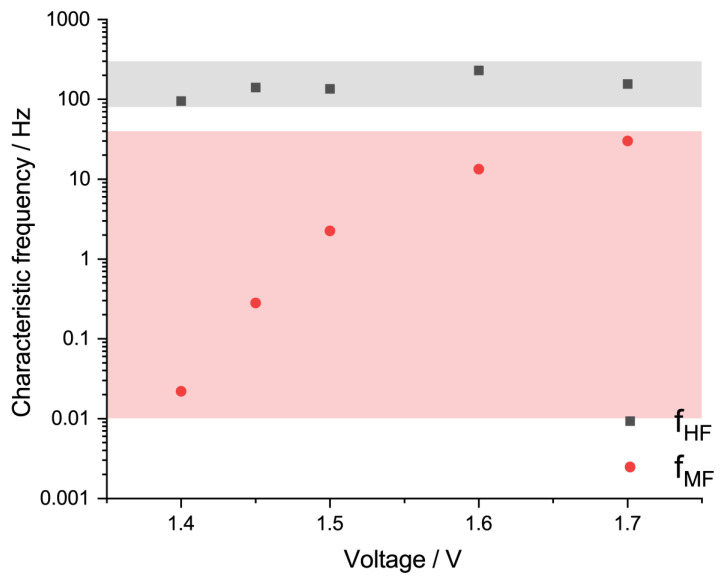
Characteristic frequencies related to the high- and mid-frequency arcs (f_HF_ and f_MF_, respectively), calculated for voltage range of 1.4–1.7 V (temperature 60 °C, 1 M KOH) (dots); filled area related to high- and mid-frequency arc is colored with black and red, respectively.

It is important to note that, according to the Nyquist plots provided in Figure 3A, it is obvious that the plots are strongly juxtaposed at higher frequencies of 0.2–1.0 kHz, suggesting the existence of a process which is independent of the voltage. Applying the approach described in [53], we provide the characteristic frequencies of the proposed processes as follows in Figure 6. The characteristic frequency f_MF_, increases with an increase in the voltage, which is typical for an electrochemical reaction, due to the decrease in its charge transfer resistance. In contrast, the f_HF_ falls to a narrow range of 80–300 Hz, suggesting its independence of the cell voltage. Such data could be useful as a reference for a Sustainion^@^-based MEA evaluated in widely used conditions (60 °C, with a 1 M KOH supporting electrolyte). It is important to note that the characteristic frequencies are provided for a limited voltage range, as at higher voltages the high-frequency and OER contributions considerably overlap, making it impossible to accurately distinguish them.

## 4. Conclusions

The detailed EIS study of an AEM water electrolyzer based on the commercially available Sustainion^®^ membrane was performed. The main electrochemical parameters of the MEA, such as ohmic and polarization resistance, were carefully evaluated, considering the proposed equivalent circuit with a managed stray impedance. The dependence of the MEA EIS footprint on the voltage (or current density) and further fitting allowed us to unravel the non-faradaic contribution at high frequencies. The decrease in KOH concentration deteriorates the AEM electrolyzer performance through both ohmic resistance and charge transfer resistance hindering the OER kinetics, likely due to decreased OH^−^ mobility. The KOH flow rate was optimized at 3 mL min^−1^ (0.4 mL min^−1^), though its effect on the MEA ohmic and charge transfer resistance depends on the operating voltage. Finally, the structure of polarization resistance was discussed, considering a negligible contribution of the HER as a Pt/C catalyst was used at the cathode.

## Data Availability

Data sharing not applicable.

## Supplementary Materials

The following supporting information can be downloaded at: https://www.mdpi.com/article/10.3390/membranes13020192/s1, Figure S1: The calculated residuals of the spectra regarding the Kramers-Kronig criteria obtained using the Lin-KK software. The raw EIS were measured at 60 °C with 1 M KOH at flow rate of 3 mL min^−1^; Figure S2: The equivalent circuit used to process the EIS spectra for “Empty” cell measurements (further referred as “stray impedance subcircuit”); Figure S3: The Bode plots of Reference measurements; Figure S4: The effect of current density on the parameters of “Empty” cell EIS: R2 and L2 (see equivalent circuit in Figure S2); Figure S5: The comparison of the raw EIS spectra, fitting curve and the simulation with the stray impedance excluded. Measured at the voltage of 1.6 V, 60 °C and 1 M KOH circulated at 3 mL min^−1^ flow rate; Table S1: Literature survey on electrolyzer performances using Sustanion^®^ membrane; Table S2: EIS fitting parameters for data given in Figure 3.
