# Supplementary material for: On the Operational Conditions’ Effect on the Performance of an Anion Exchange Membrane Water Electrolyzer: Electrochemical Impedance Spectroscopy Study
Source: Membranes (Basel). 2023 Feb 3;13(2):192. doi: 10.3390/membranes13020192 (PMC9966502; doi:10.3390/membranes13020192)
Supplement: Supplementary file 1 [file membranes-13-00192-s001.zip › membranes-2157611-supplementary.pdf]

Supporting information

On the Operational Conditions' Effect on the Performance of an Anion Exchange Membrane Water Electrolyzer: Electrochemical Impedance Spectroscopy Study

Irina V. Pushkareva [1,2], Maksim A. Solovyev [1,2], Sergey I. Butrim [1,2], Margarita V. Kozlova [1,2], Dmitri A. Simkin [1] and Artem .S. Pushkarev [1,2,*]

[1] National Research Center "Kurchatov Institute", 1 Kurchatov sq., Moscow 123182, Russia
[2] National Research University "Moscow Power Engineering Institute", Dept. of Chemistry and Electrochemical Energy, 14 Krasnokazarmennaya str., Moscow 111250, Russia
* Correspondence: pushkarev_as@outlook.com

Lin-KK [1] software was used to evaluate the quality of measured EIS data of the AEM water electrolyzer. The calculated residuals of the raw spectrums in regard to the Kramers-Kronig criteria is shown in Fig. 1S. These are consistently very low and thus indicate valid impedance spectra in compliance with the linearity and time invariance (LTI) criteria [2]. Important to note, that residuals are generally less than 2%, except for points at 100 and 200 Hz, which are considered to be artefacts. Higher residuals at higher frequencies comes from stray impedance discussed further in SI The stray impedance is rather hard to be processed by the Lin-KK due to its algorithm giving higher residuals value.

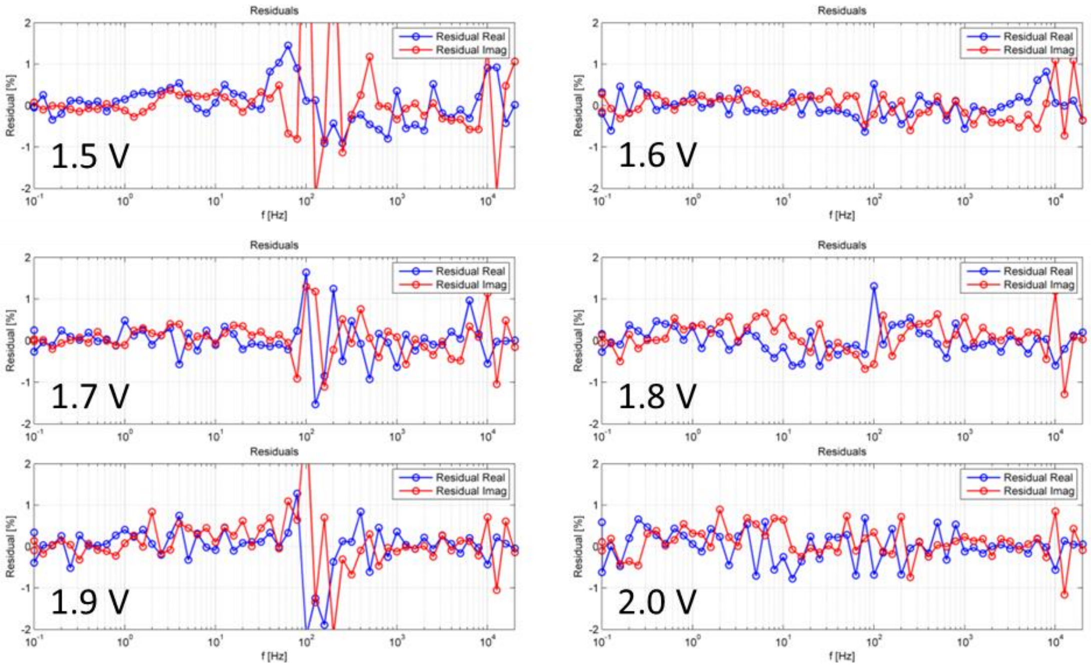

Figure S1. The calculated residuals of the spectra regarding the Kramers-Kronig criteria obtained using the Lin-KK software. The raw EIS were measured at 60 °C with 1M KOH at flow rate of 3 ml min^{-1}.

The contribution of the hardware and test bench (cables, cell connections, etc.) used for in-situ measurements except from those originating from the CCM should be analyzed to avoid misinterpreting of the EIS spectra [3]. For instance, the MEA ohmic resistance (also referred as high frequency resistance, if taken simply from the EIS spectra intersection with real impedance axis at high frequencies [4]) usually assessed at high frequencies of 1–25 kHz [5,6]. HFR could be

significantly affected by stray impedance coming from test bench and equipment, as it mainly effects the MEA EIS footprint at high frequencies (high frequency "hook", Fig. 4S).

The procedure to access the external influence on the water electrolysis EIS (referred as Reference measurement) was based on approach described in Agilent Impedance Measurement Handbook [7] and shortly in Ref. [8], and was performed as follows. The test cell setup consists of Ni foam PTL only sandwiched between flow fields (short circuit connection c) instead of MEA. The response of this setup under different current densities is characterized via EIS in galvanostatic mode (amplitude 5%, 20 kHz – 10 Hz). The EIS measurements of shortened test cell in potentiostatic mode were noisy and distorted, and could be stabilized only by relatively high amplitude – up to 20 mV at voltage range 0.05 – 0.15 V. So, the galvanostatic mode was chosen for Reference measurements, and the equivalent circuit is given Fig. 2S. The Bode plots of Reference measurements are given in Fig. 3S.

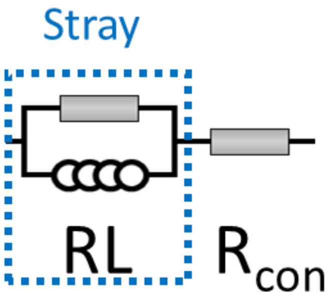

Figure S2. The equivalent circuit used to process the EIS spectra for "Empty" cell measurements (further referred as "stray impedance subcircuit").

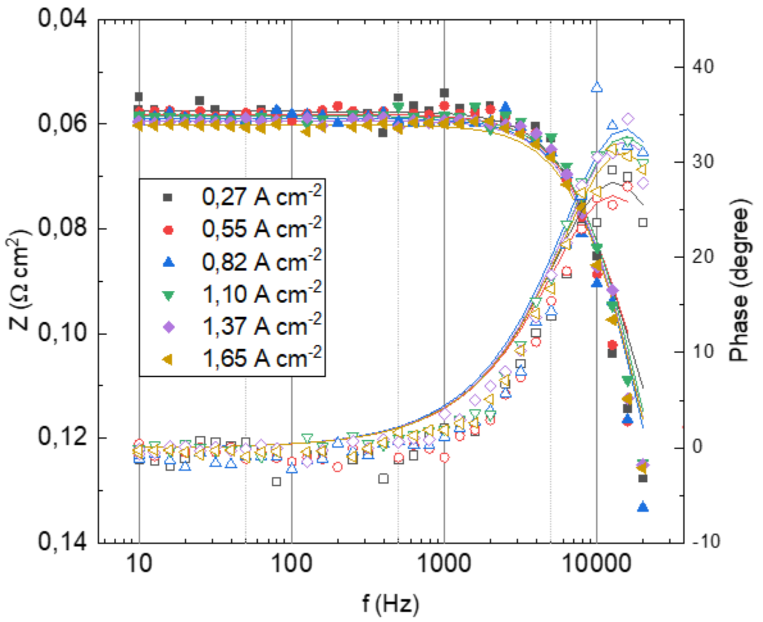

Figure S3. The Bode plots of Reference measurements.

Obviously, the effect of current density on the inductive component L_2 is almost negligible in wide range of 0.25–1.75 A cm^{-2} (Fig. 4S). Important to note that the particular cell connection affects

the value of L_2, so its value variation is in the narrow gap of *ca.* 0.8–1.2·10^{-7}, which is used for the fitting of MEA equipped test cell spectrums. In contrast, R_2 variation is rather high in comparison to the MEA ohmic resistance (*ca.* 0.01 Ω), so this parameter is used as a ”free” one, though the values obtained in Reference measurements are used as initial.

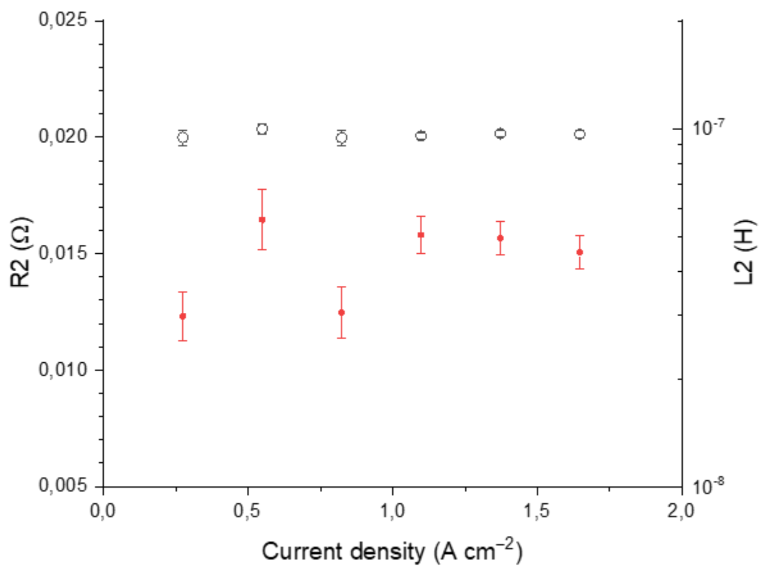

Figure S4. The effect of current density on the parameters of “Empty” cell EIS: R2 and L2 (see equivalent circuit in fig. 2S).

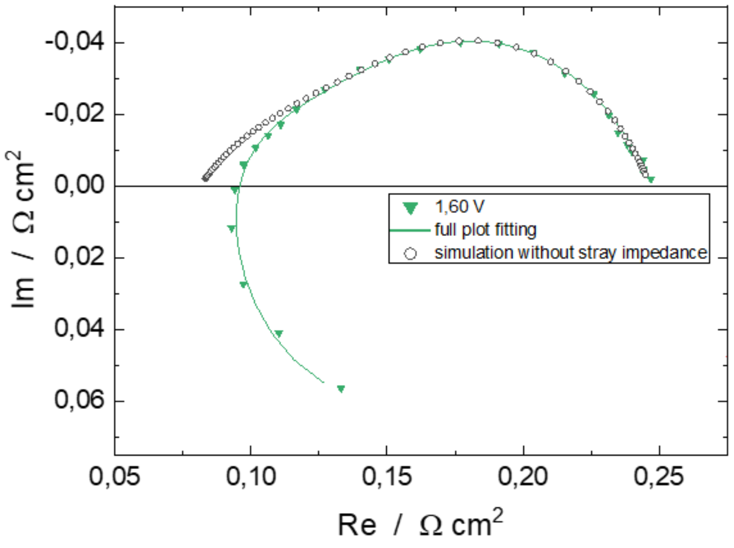

Figure 5S. The comparison of the raw EIS spectra, fitting curve and the simulation with the stray impedance excluded. Measured at the voltage of 1.6 V, 60 °C and 1M KOH circulated at 3 ml min^{-1} flow rate.

Table S1. Literature survey on electrolyzer performances using Sustanion® membrane.

Membrane Grade	Anode		Cathode		Feeding approach	MEA preparation approach[1]	Performance			Ref.
	catalyst (binder)	PTL type	catalyst (binder)	PTL type			Voltage, V	Current density, A/cm^2	T, °C	
Sustainion X37-50 (Dioxide Materials, US)	NiO (3 mg/cm^2) and Nafion binder	Ni foam	Pt/C (0.8 mg_{Pt}/cm^2 (PM40, PROMETHEUS R&D, LLC, Russia) and Nafion binder	Ni foam	1 M KOH Anode and cathode flow rate is 3 ml/min	CCS	1.74	1	60	This work
Sustainion X37-50	$NiFe_2O_4$ (1.8 mg/cm^2) (US Research Nanomaterials, US)	Stainless-steel GDL (Dioxide Materials, US)	Modified Raney nickel (2.7 mg/cm^2) (Sigma Aldrich)	Nickel fiber paper (Dioxide Materials)	1M KOH, evenly split to both the anode and cathode, **total** pump flowrate is about 10 mL/min	CCS	1.8	0.744	60	[10]
Sustainion Grade T	$NiFe_2O_4$ (1.8 mg/cm^2) (US Research Nanomaterials, US)	Stainless-steel GDL (Dioxide Materials, US)	Modified Raney nickel (14.5 mg/cm^2) (Sigma Aldrich)	Nickel fiber paper (Dioxide Materials)	1M KOH, evenly split to both the anode and cathode, **total** pump flowrate is about 10 mL/min	CCS	1.8	0.837	60	[10]
Sustainion XB-37	Co (Alfa Aesar) and Sustainion XB-7 binder (0,4 mg/cm^2, ionomer:	Carbon Toray paper	47% Pt/HSC (Tanaka Kikinzoku Kogyo) and Sustainion XB-7 binder (0.1	Toray carbon paper	1M KOH, Anode and cathode flow rate is 100 ml/min	CCS	1.72	0.5	60	[11]

[1] CCM – catalyst coated membrane; CCS – catalyst coated substrate

	catalyst ratio is 0.22)		mg_{Pt}/cm^2, ionomer: catalyst ratio is 0.44)							
Sustainion X37-50 grade T	$NiFeCoO_x$ (25 mg/cm^2) and Nafion binder (10 wt.% of the catalyst amount)	Ni foam	40% Pt/C (HISPEC 4000, Johnson Matthey, London, UK) Nafion binder (20 wt.% of the catalyst amount)	carbon paper (Sigracet 29BC)	1M KOH, 90 ml/min (only anode side)	CCS	1.9	1	70	[12]
Sustainion X37–50	$NiFe_2O_4$ (Sigma Aldrich) (2 mg/cm^2) and Nafion binder	316L sintered stainless steel fiber felt (Bekaert)	NiFeCo (US Nano) (2 mg/cm^2) and Nafion binder	Sigracet 39BC GDL	1 M KOH was recirculated through the anode and cathode at a rate of 2 ml/min	CCS	1.9	1	60	[13]
PTFE-reinforced Sustainion X37-50 grade T	IrO_2 (2.0 mg/cm^2, Alfa Aesar, MA, USA) and 10 wt.% PTFE binder	Titanium felt (Bakaert, Belgium)	46.6% Pt/C (0.5 mg/cm^2, TANAKA Co., Japan) and 10 wt.% PTFE binder	carbon paper (Sigracet 39 BC, SGL carbon, Germany)	1M KOH solution into the anode at a flow rate of 15–35 mL/min	CCS	2	2	60	[14]
PTFE-reinforced Sustainion X37-50 grade T	RANEY® Ni–Fe (20 mg/cm^2)	Ni foam (Alantum, Germany)	RANEY® Ni–Fe (20 mg/cm^2)	Ni foam (Alantum, Germany)	1M KOH solution into the anode at a flow rate of 15–35 mL/min	CCS	2	0.62	60	[14]
Sustainion X37-50 grade T	NiFe-LDH (2.5 mg/cm^2)/ Ketjen black (EC600JD, Lion Specialty Chemicals) and Nafion binder	carbon paper (TGP-H-120, Toray)	Pt/C (1.3 mg/cm^2, TEC10E50E, Tanaka Kikinzoku Kogyo) and Nafion binder	carbon paper (TGP-H-120, Toray)	1M KOH	CCS	1.67	1	60	[15]
Sustainion X37-50 grade T	IrOx (1.9 mg/cm^2) and Nafion binder	carbon paper (TGP-H-120, Toray)	Pt/C (1.2 mg/cm^2, TEC10E50E, Tanaka	carbon paper (TGP-H-120, Toray)	1M KOH	CCS	1.67	1	60	[15]

			Kikinzoku Kogyo) and Nafion binder							
Sustainion X37-50 grade T	NiMo-NH_3/H_2	Carbon paper	Fe-NiMoNH_3/H_2	Carbon paper	1M KOH	CCS	1.57	1	80	[16]
Sustainion X37-50 grade T	NiMo-NH_3/H_2	Carbon paper	Fe-NiMoNH_3/H_2	Carbon paper	1M KOH	CCS	1.62	1	60	[16]

Table S2. EIS fitting parameters for data given in Fig. 3.

Parameter	1.4 V	1.45 V	1.5 V	1.6 V	1.7 V	1.8 V	1.9 V	2.0 V
R_1, mΩ cm^{-2}	80.7±0.7	79.1±0.5	79.9±0.5	81.2±0.5	79.6±0.6	79.1±0.5	78.0±0.5	79.6±0.7
R_2,	155.8±6.9	126.8±3.7	129.0±3.7	127.7±3.1	134.4±3.4	149.0±2.7	133.0±2.0	133.8±1.9
L_2, H	$9.6\cdot10^{-8}$	$9.65\cdot10^{-8}$	$9.64\cdot10^{-8}$	$9.68\cdot10^{-8}$	$9.8\cdot10^{-8}$	$11.0\cdot10^{-8}$	$11.7\cdot10^{-8}$	$11.4\cdot10^{-8}$
R_3, mΩ cm^{-2}	93.0±2.4	90.9±2.0	90.0±3.5	70.2±9.4	86.2±41.1	93.2±3.1	81.7±3.1	74.6±4.8
Q_3	2.72±0.10	2.27±0.07	2.34±0.08	2.26±0.09	2.25±0.10	2.37±0.28	2.29±0.30	2.18±0.42
α_3	0.526[2]							
R_4, mΩ cm^{-2}	18517±1095	1636±18	338±5	95.4±10.0	26.1±39.1	4.0±3.7	4.6±3.6	5.7±5.1
Q_4	2.25±0.02	2.82±0.03	2.31±0.05	2.81±0.2	3.78±1.65	0.16±0.34	0.11±0.2	0.1±0.16
α_4	0.89	0.82	0.84	0.74	0.82	1	1	1
R_5, mΩ cm^{-2}	–	–	–	–	6.5±3.2	5.9±1.8	7.6±1.3	11.0±1.3
Q_5	–	–	–	–	314±149	331±99	148±28	90±12
α_5	–	–	–	–	1	1	1	1

The sharp decrease and high variation of Q4 values at voltage ≥1.8 V could be explained by strong overlapping of the R3/CPE3 and R4/CPE4 due to the increased reaction rates at high voltage in comparison with non-faradaic contribution. So, it is not straightforward to distinguish faradaic and non-faradaic contributions when their charge transfer rates are rather similar. The application of alternative approaches of EIS processing [17].

[2] The fixed α value were chosen according to our recent findings on the non-faradaic contributions in AEM WE impedance [Pushkarev A.S. et al. PGM-free electrocatalytic layer characterization by electrochemical impedance spectroscopy of an anion exchange membrane water electrolyzer with Nafion ionomer as the bonding agent // Catalysts. 2023] and considering the shape of Nyquist plots at high frequencies (see Fig. 3 of the paper), which is independent of the current density.

References

1. Schönleber M., Klotz D., Ivers-Tiffée E. A Method for Improving the Robustness of linear Kramers-Kronig Validity Tests // Electrochim. Acta. 2014. Vol. 131. P. 20–27.
2. Zappen H. et al. In-Operando Impedance Spectroscopy and Ultrasonic Measurements during High-Temperature Abuse Experiments on Lithium-Ion Batteries // Batteries. 2020. Vol. 6, № 2. P. 25.
3. Shin E.-C. et al. Polarization mechanism of high temperature electrolysis in a Ni–YSZ/YSZ/LSM solid oxide cell by parametric impedance analysis // Solid State Ionics. 2013. Vol. 232. P. 80–96.
4. Suermann M., Bensmann B., Hanke-Rauschenbach R. Degradation of Proton Exchange Membrane (PEM) Water Electrolysis Cells: Looking Beyond the Cell Voltage Increase // J. Electrochem. Soc. 2019. Vol. 166, № 10. P. F645–F652.
5. Suermann M. et al. High pressure polymer electrolyte water electrolysis: Test bench development and electrochemical analysis // Int. J. Hydrogen Energy. 2017. Vol. 42, № 17. P. 12076–12086.
6. Holzapfel P. et al. Directly coated membrane electrode assemblies for proton exchange membrane water electrolysis // Electrochem. Commun. 2020. Vol. 110. P. 106640.
7. Agilent Impedance Measurement Handbook. 4th Editio. Agilent Technologies, Inc., 2009. 140 p.
8. Meddings N. et al. Application of electrochemical impedance spectroscopy to commercial Li-ion cells: A review // J. Power Sources. 2020. Vol. 480. P. 228742.
9. Papakonstantinou G. et al. Degradation study of a proton exchange membrane water electrolyzer under dynamic operation conditions // Appl. Energy. 2020. Vol. 280. P. 115911.
10. Motealleh B. et al. Next-generation anion exchange membrane water electrolyzers operating for commercially relevant lifetimes // Int. J. Hydrogen Energy. 2021. Vol. 46, № 5. P. 3379–3386.
11. Ghoshal S., Pivovar B.S., Alia S.M. Evaluating the effect of membrane-ionomer combinations and supporting electrolytes on the performance of cobalt nanoparticle anodes in anion exchange membrane electrolyzers // J. Power Sources. 2021. Vol. 488. P. 229433.
12. Ahmed K.W. et al. NiFeOx and NiFeCoOx Catalysts for Anion Exchange Membrane Water Electrolysis // Electrochem. 2022. Vol. 3, № 4. P. 843–861.
13. Liu Z. et al. The effect of membrane on an alkaline water electrolyzer // Int. J. Hydrogen Energy. 2017. Vol. 42, № 50. P. 29661–29665.
14. Chen N. et al. High-performance anion exchange membrane water electrolyzers with a current density of 7.68 A cm −2 and a durability of 1000 hours // Energy Environ. Sci. 2021. Vol. 14, № 12. P. 6338–6348.
15. Koshikawa H. et al. Single Nanometer-Sized NiFe-Layered Double Hydroxides as Anode Catalyst in Anion Exchange Membrane Water Electrolysis Cell with Energy Conversion Efficiency of 74.7% at 1.0 A cm –2 // ACS Catal. 2020. Vol. 10, № 3. P. 1886–1893.

16. Chen P., Hu X. High-Efficiency Anion Exchange Membrane Water Electrolysis Employing Non-Noble Metal Catalysts // Adv. Energy Mater. 2020. Vol. 10, № 39. P. 2002285.

17. Ciucci F. Modeling electrochemical impedance spectroscopy // Curr. Opin. Electrochem. 2019. Vol. 13. P. 132–139.
